# Quality by design optimisation of isothermal dry particle coating for enhanced buccal permeation of vancomycin

**DOI:** 10.1038/s41598-025-29164-2

**Published:** 2025-11-25

**Authors:** Anthony Rajabi, Affiong Iyire, David Wyatt, Jasdip Koner, Afzal R. Mohammed

**Affiliations:** 1 Aston Pharmacy School, College of Health and Life Sciences, Aston University, Birmingham, B4 7ET UK; 2 Aston Particle Technologies Ltd, Birmingham, UK

**Keywords:** Buccal delivery, Quality by design, Ion pair, Vancomycin, Biologics, Permeation, Dry particle coating, Peptide delivery, Drug development, Biomedical engineering, Drug delivery

## Abstract

The formulation and manufacture of macromolecules for oral delivery present persistent challenges owing to high molecular weight, pH sensitivity and manufacturing complexity. Consequently, over 90% of FDA-approved biologics are administered by invasive methods. Buccal delivery offers a promising non-invasive alternative, as it bypasses first-pass metabolism, avoids gastrointestinal degradation, and can improve patient compliance. Here we evaluate isothermal dry particle coating (iDPC) as a scalable, solvent-free approach to enhance buccal permeation by forming ion-pair coatings on drug particles. In iDPC, centrifugal and gas-drag forces promote systematic collisions between host and guest particles, here vancomycin and L-glutamic acid, yielding uniform surface coverage that facilitates buccal permeation. This study utilised a Design of Experiments (DoE) methodology within a Quality by Design (QbD) framework to optimise iDPC processing for vancomycin, a Biopharmaceutics Classification System (BCS) Class III glycopeptide with poor oral bioavailability. A Central Composite Face (CCF) design was utilised to investigate the interactive effects of five critical process parameters (CPPs): pre-processing time, processing time, nitrogen flow rate, drum speed and amino acid concentration, on two critical quality attributes (CQAs): content uniformity and 60-minute permeation across TR146 buccal epithelium. Regression modelling identified that increases in L-glutamic acid concentration and drum speed were the key factors enhancing permeation, while processing time and drum speed were the key variables improving content uniformity. A predictive 4D design space identified operating regions with a high probability of simultaneously meeting prespecified targets (permeation ≥ 40% and RSD ≤ 5%). The models demonstrated strong fit (R^2^ = 0.767 for permeation; 0.774 for content uniformity), with non-significant lack-of-fit, and performance improved markedly, with content uniformity ranging from 0.93 to 6.29% RSD and permeation increasing from 36% to 60% under optimised conditions. Mechanistic analysis indicated that drag from the nitrogen curtain impacted the fluidisation of cohesive L-glutamic acid fine particles, while total energy input promoted deagglomeration and dispersion, thereby improving uniformity. These findings demonstrate that iDPC is a robust manufacturing approach for buccal delivery of biologics, providing controlled particle level modification without the use of solvents. The QbD-driven DoE establishes clear links between CPPs and CQAs, supports the development of control strategies, and provides a basis for regulatory flexibility in the non-invasive delivery of large molecule therapeutics.

**Supplementary Information:**

The online version contains supplementary material available at 10.1038/s41598-025-29164-2.

## Introduction

The use of biologics has revolutionised the treatment for numerous diseases, primarily due to their superiority in terms of specificity and potency in treating a wide range of severe and chronic conditions^1,2^. As a result, eight out of the top ten best selling drugs in 2024 were biologics^1–4^. Biologics encompass a broad spectrum of medicines including but not limited to, peptides, vaccines, monoclonal antibodies, and nucleic acids^2^. However, a notable drawback associated with biologics is the reliance on invasive delivery methods, which may require stringent storage conditions, administration by medical personnel and results in patient discomfort^5^.

Biologics, characterised by their large, complex molecular structure, exhibit poor oral bioavailability attributed to their size, susceptibility to enzymatic degradation and hydrophilic nature, which collectively limit their permeation across epithelia^5^. In addition, the inherent chemical and physical instability of biologics poses significant challenges when formulating non-invasive drug delivery systems. These molecules are highly sensitive to factors such as temperature, pH variations, shear forces and chemical degradation, resulting in alterations in therapeutic activity and potentially triggering immunogenic responses^6,7^.

As a result, both academia and industry have prioritised the development of non-invasive delivery systems capable of preserving the safety and therapeutic efficacy of biologics^1,5^. Among the non-invasive routes of administration, the buccal route offers various advantages including avoidance of gastrointestinal degradation, bypassing first-pass metabolism, and enhanced patient acceptability^8^.

The Quality by Design (QbD) approach, advocates that product quality, in regards to both safety and efficacy, is built into the product during manufacture, rather than solely relying on increased testing of the final product^9^. In pharmaceutical contexts, QbD is defined as “a systematic approach to development, that begins with predefined objectives, and emphasises product-process understanding and control based on sound science and quality risk management”^10^. Central to the pharmaceutical QbD framework are several key elements: The Quality Target Product Profile (QTPP), CQA, and CPPs^10,11^.

The QbD process initiates with defining the QTPP, which serves as a prospective synopsis of the quality attributes essential for achieving the desired product quality, while also considering the safety and efficacy of the product^10,12^. Through the QTPP, CQAs are identified, representing the physical, chemical, biological, or microbiological properties crucial for ensuring the desired product quality^10,11,13^. Subsequently, conducting a comprehensive risk assessment, as outlined in ICH Q9 guidelines, facilitates the identification of the CPPs directly influencing the CQAs^14^. The CPPs are pivotal variables that significantly influence the quality of the final product and inform the DoE^12,14^. The DoE, a structured and organised methodology, determines the relationships between CPPs and their effects on CQAs, through the establishment of mathematical models^12,15,16^.

Implementing a QbD approach in formulating non-invasive biologics can yield significant advancements across various key categories^17^. By adopting a QbD framework, insights into both the biologic and the excipients utilised can enable a deeper understanding of how the materials will interact within the process. This comprehensive understanding facilitates the evaluation of potential implications on the biologics, thereby improving product consistency and safety profile^18^.

This is particularly important in biologic formulation development, where deviations in CPPs, such as temperature, may occur during manufacturing. Through QbD, a predefined design space can be established, allowing for the determination of tolerable limits for such deviations. This mitigates uncertainties regarding the formulation’s safety profile and therapeutic efficacy, ensuring the formulation’s integrity^19^. In addition, QbD principles extend beyond the development phase, encompassing the entire product lifecycle. Continuous monitoring and improvement are integral elements of QbD, which enables adjustment in response to new knowledge and techniques; this ongoing optimisation can improve the performance and effectiveness of the product^18^.

This paper provides a holistic assessment of the impact of process parameters in isothermal dry particle coating using amino acids as counter ions to deliver large molecule drugs and study buccal permeability^20^. The model compound chosen for this study was vancomycin, a BCS Class III drug, characterised by its oral bioavailability of less than 10%^21,22^. The formulation strategy involved utilising an iDPC. The iDPC mechanism utilises a high-speed rotating vessel in conjunction with a fluidising nitrogen gas blade, resulting in a unique thin-layer fluidisation mechanism which processes powders in a solvent-free, ambient, and dry state^8^. iDPC does not require prior mechanical mixing of the drug with the excipient; coating forms in situ under centrifugal force and gas drag, minimising mechanical stress on the powder bed and preserving the integrity of biologics sensitive to shear. According to manufacturer guidelines, the guest particles must be at least 2–3 times smaller than the host particles to ensure effective and uniform coating. In this study the guest-host size disparity was selected to favour a uniform, near-monolayer coating of L-glutamic acid onto vancomycin, maximising the potential for ion-pair interactions. The effect of varying L-glutamic acid particle size was not evaluated; however, we hypothesise that larger guest particles would reduce surface coverage, diminish ion-pair formation and consequently reduce permeation.

In this study, the coating process was investigated by varying critical process parameters that are associated with the iDPC: flow rate, process time, and centrifugal force. Flow rate creates a fluidised environment that promotes collisions between smaller and larger particles, thereby enhancing both blending and coating^23^. Process time signifies how long the powders remain in the rotating drum, allowing sufficient particle interactions to improve coating uniformity for different materials. Centrifugal force, determined by drum speed, aids deagglomeration, allowing for greater particle-to-particle contact, thereby achieving a more uniform coating^8^. Together, these parameters can determine the extent and homogeneity of the coating and therefore impact the pharmaceutical application.

The objective of this study was to apply a QbD-approach to optimise key process parameters associated with the iDPC, with the aim of identifying and understanding the design space and to assess how coating vancomycin particles with L-glutamic acid affects vancomycin permeation through the TR146 buccal epithelial cell line. A Response Surface Model (RSM) was employed to evaluate the effects of varying interactive terms among the CPPs (pre-processing time, processing time, drum speed, airflow, and amino acid concentration) on two key CQAs: content uniformity and vancomycin permeation percentage at 60 min. The resulting model was used to establish a predictive design space for the iDPC process.

## Results and discussion

### Screening studies

In previous work, a screening study was conducted to gain insight into how weakly basic and acidic amino acids would impact the permeation profile of vancomycin over a 60-minute period^8,20^. Based on the results, L-glutamic acid was selected as the amino acid for this study. Subsequently, a risk assessment was undertaken to identify the CPPs with the highest impact on the CQAs. These CPPs were then integrated into an optimisation study utilising a DoE approach based on a five-factor, multi-level CCF design plan. Vancomycin was selected as the model compound; a BCS Class III glycopeptide macromolecule, with an oral bioavailability of less than 10% and is therefore primarily given invasively^21^. The Ion-pair strategy involves combining an ionisable drug with a counter ion, forming a more lipophilic, neutral complex held together by Coulombic attractions, which enhance the permeability across cell membranes^24^. Amino acids have been identified as safe counter ions capable of aiding the permeation of large molecules^8,20^. In previous work, L-glutamic acid and L-histidine were tested in an initial screening permeation study to determine their effectiveness in enhancing the permeability of vancomycin across the buccal cell line, TR146^8,20^. The selection of L-glutamic acid and L-histidine was based on their specific pKa values, as these values play a crucial role in their ability to form ion-pair complexes with vancomycin. The formulation containing vancomycin and L-glutamic acid demonstrated a significant increase in vancomycin that permeated the TR146 buccal cell layers compared to the control (ANOVA, *P* = 0.0022), whereas the formulation containing vancomycin and L-histidine did not achieve a significant difference in vancomycin permeation (ANOVA, *P* = 0.0548). Based on the enhanced permeation profile of vancomycin formulations containing L-glutamic acid, it was selected as the amino acid for this study^8^.

### Application of QbD principles for process optimisation

Following the selection of L-glutamic acid, the process variables: pre-processing time, processing time, flow rate, and relative centrifugal force were selected for optimisation within the iDPC. The responses obtained from the screening experiment were vancomycin content uniformity, and the permeation percentage of vancomycin through the TR146 buccal cell line over a duration of 60 min. The optimisation of the process included establishing the specifications for the responses based on risk assessment; and conducting a DoE study.

### Risk assessment for CQAs and process variables

The risk assessment was conducted to assess the influence of the amino acid concentration and the iDPC process parameters on the CQAs, facilitating the identification of independent factors utilised in the DoE experimental design. Risk ranking was determined based on compendia requirements, initial screening results or literature review. Process parameters with risk rankings ranging between medium and high were taken as factors in the DoE. The results of the risk assessment can be seen in Table 1.

**Table 1:** Risk assessment including the justification of the input and process parameters against the CQAs and the justification for the risk ranking.

Quality attributes/process variables	Content uniformity	Permeation %	Justification
Pre-process time (minutes)	Medium	Medium	Nitrogen flow rate, speed, and processing times directly influence the blend homogeneity^25–27^. The concentration of L-glutamic acid directly impacts ion-pair formation with vancomycin, consequently affecting the permeation profile^28,29^
Process time (minutes)	High	High	
Flow rate (l/min)	High	High	
Speed (rpm)	High	Medium	
L-glutamic acid concentration (%)	Medium	High	

### Critical quality attributes (CQAs)

The CQA, content uniformity was identified by the acceptable values set out by the British Pharmacopoeia and United States Pharmacopoeia^30,31^. The second CQA, permeation through the TR146 buccal cell line was identified by literature review of acceptable methods for assessing buccal permeation^32,33^. The list of CQAs, seen in Table 2, were identified from the QTPP based on the impact of each parameter on the production of buccal formulations.

**Table 2:** Summary of targeted outcome and implications for the CQAs, content uniformity and permeation percentage.

CQA (Responses)	Targeted outcome	Implications
Content uniformity	Relative standard deviation (RSD) $\le$ 5%, aligning with British Pharmacopoeia and the United States Pharmacopeia^30,31^	Deviations from the required outcome directly indicate formulation failure
Permeation percentage through TR146 buccal cell line	Percentage release of API at 60 min is greater than the control (unformulated vancomycin)	Failure to enhance the permeation profile of unprocessed vancomycin indicates process failure

### Model verification

Following the selection of process variables and responses (CPPs and CQAs, respectively), the DoE software, MODDE, developed the candidate model as a quadratic process model, with 29 experiments that were carried out according to the randomisation order set by the software, of which three experiments, known as centre points, represented replicates necessary to ensure model reproducibility. The compiled worksheet was then fitted into the quadratic process model. Once the model was fitted, model verification and regression equations enabled detailed understanding of the impact of the process parameters on the responses.

**Fig. 1:**
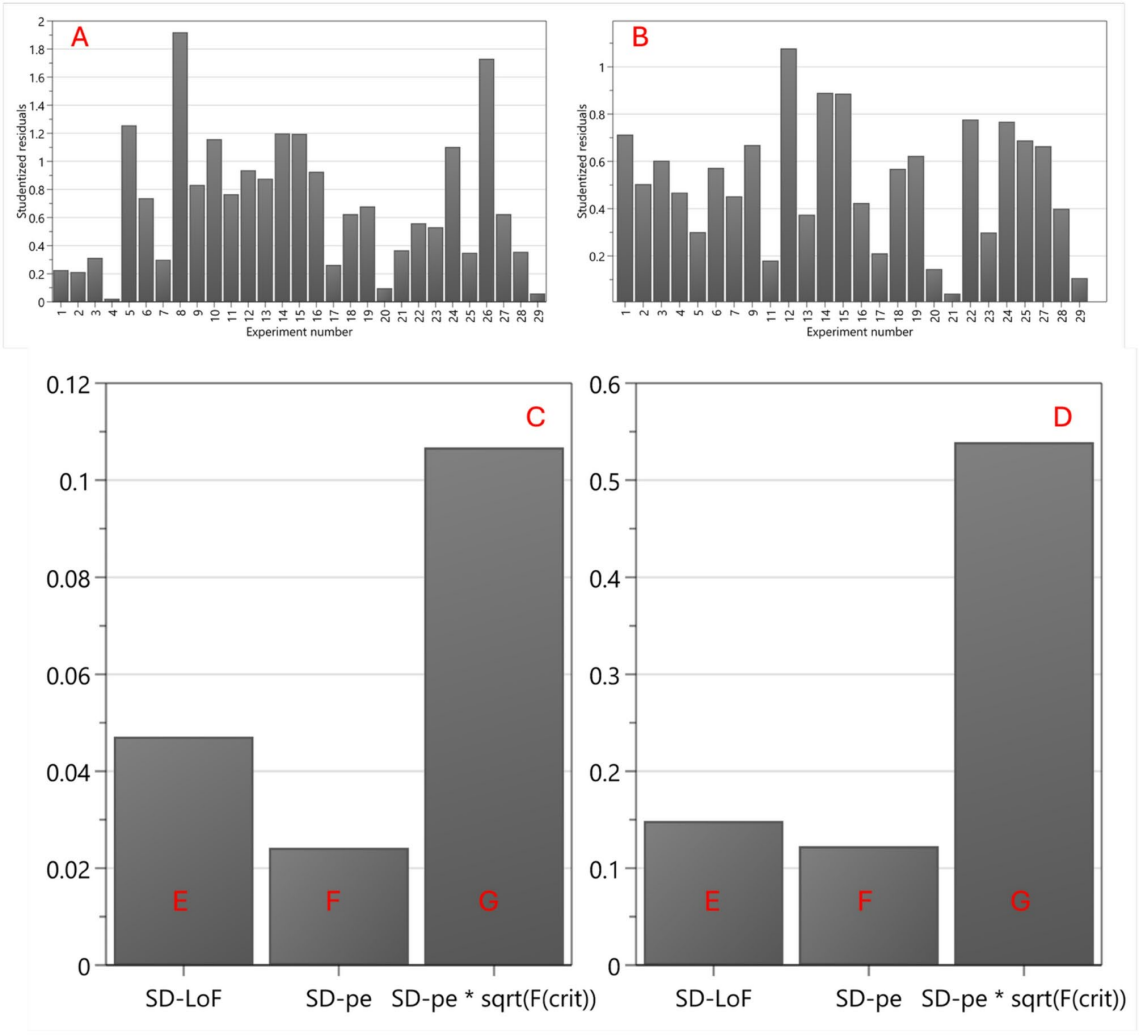
(**A**) The distance to model plot with all experiment runs included, (**B**) is the distance to model plot following the removal of experiment number 8, 10, and 26, illustrating a reduced variation in the model. (Below) Lack of fit plot showing standard deviation (SD) due to lack of fit (**A**), SD of pure error (**B**) and SD of pure error * critical F-value (**C**) for the two responses. If A ≤ C, the model shows no lack of fit. (**C**) Shows the lack of fit plot for % permeation. (**D**) shows the lack of fit plot for content uniformity.

**Fig. 2:**
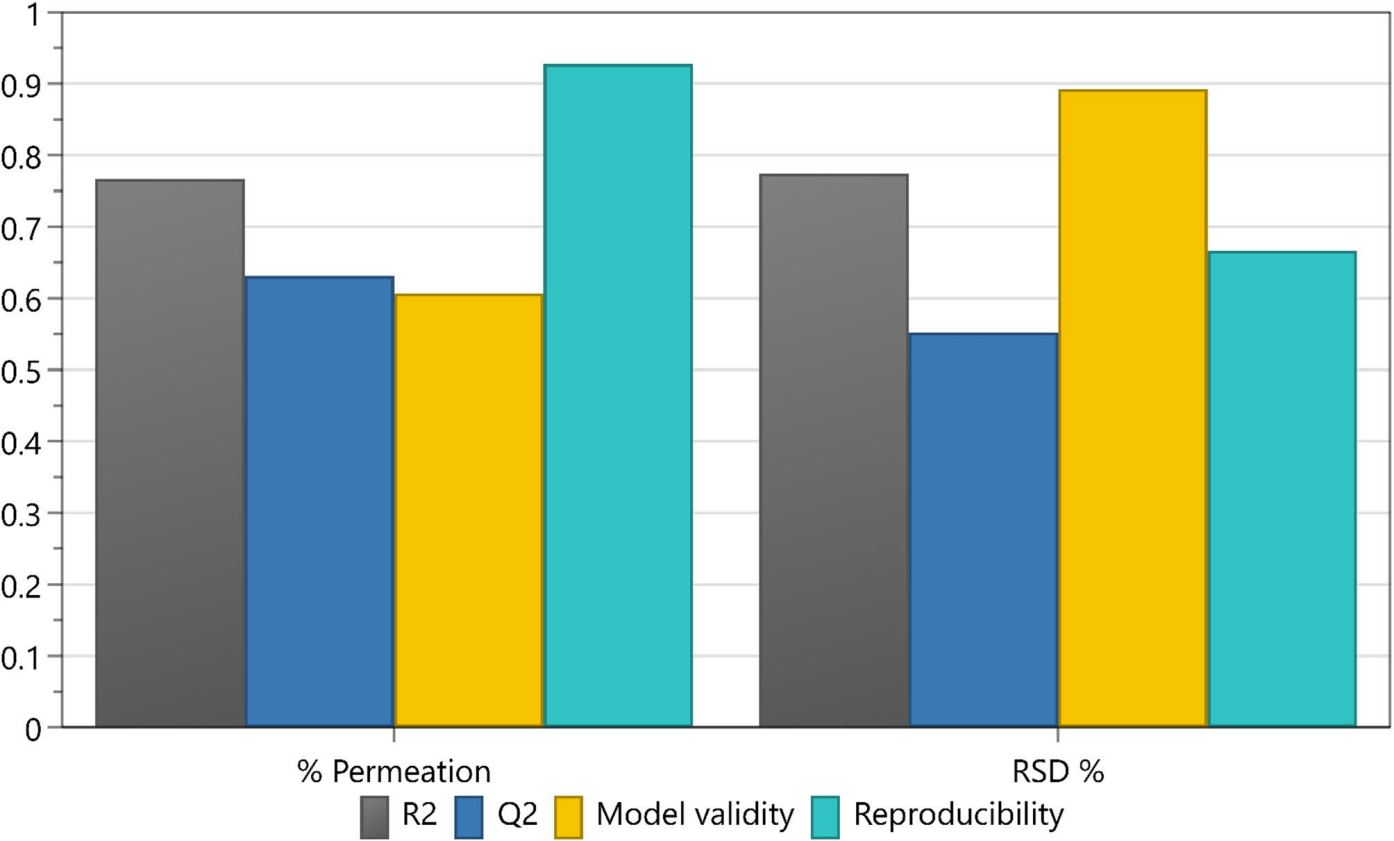
A summary of fit plot of the two CQAs of fit showing model fit (R2), predictability (Q2), validity, and reproducibility. Model fitted with Partial Least Squares (PLS).

The model was verified by utilising a combination of the distance to model plot, lack of fit plot (Fig. 1A–D) and summary of fit plot (Fig. 2). A total of 24 runs were included and 5 excluded. Experiment number 8 was the furthest from the model, followed by number 10 and then number 26. The difference in distance to model plot, following the removal of experiments numbered 8, 10 and 26 can be seen in Fig. 1A and B. To further improve the summary of fit plot, experiments 14 and 15 were removed.

Following the removal of outliers, model fitness was investigated by using the lack of fit plot, seen in Fig. 1C and D. Runs that showed large deviations from the model and were flagged as statistical outliers by the software’s diagnostic tools were excluded during model fitting. These exclusions were made on statistical grounds only and did not reflect technical failure. All excluded runs are reported in the supplementary material, Table S1. The lack of fit plot is separated into three bars, labelled E, F, and G. Bar E represents the SD-LoF, and this shows the variation of the response due to the lack of fit of the model. Bar F represents the SD-pure error, and this shows the variation due to the replicated experiments. Bar G represents SD-pe*sqrt(F(crit), which shows the SD pure error (bar F) multiplied by the square root of the critical F-value at the 95% confidence interval. To confirm model fitness, the value associated with the standard deviation due to lack of fit (bar E) must be less than the standard deviation*F-value (bar G), which is shown for both responses, Fig. 1C and D^34^.

The summary of fit plot (Fig. 2) serves as a fundamental overview of model statistics and is composed of four primary components: R2, Q2, model validity, and reproducibility. R2 indicates the model linearity, with a value below 0.5 indicating low significance. Q2 provides an estimation of future prediction precision, where a value greater than 0.1 suggests a significant model, while exceeding 0.5 indicates a good model. Furthermore, the disparity between R2 and Q2 should ideally be less than 0.3 for a good model^12,34^.

Model validity serves as a test for various model issues, with a value less than 0.25 indicating statistically significant problems such as outliers, an incorrect model specification, or issues with transformations. A low value in this metric might also suggest the absence of terms like interactions or squared terms^34^. The final component, reproducibility quantifies the variation among replicates concerning overall variability and is expected to exceed a value of 0.5 for satisfactory reproducibility.

From Fig. 2, the model exhibited a good fit across all four responses, with R2 values exceeding 0.7 for both permeation percentage and content uniformity. The predictive power for the two responses exhibited a difference of less than 0.3 to the R2 values, indicating a good model^34^. The validity of both responses was confirmed by achieving values greater than 0.5, underscoring the reliability of the models in accurately representing the underlying processes^34^. The reproducibility values for both responses were notably high, achieving values of greater than 0.6. These values suggest control over experimental conditions and minimal pure error, further reinforcing the credibility of the experimental outcomes^34^.

### Analysis of variance (ANOVA) results

Table 3 summarises the ANOVA analysis results obtained from MODDE, the p-values were < 0.05 for both CQAs, indicating that the regression model was significant for all responses. In addition, the model had no lack of fit as the *p*-values for both responses were greater than 0.05, indicating the lack of fit was insignificant.

**Table 3:** Summary of results obtained from ANOVA of the two CQAs to test model validity.

	*P*	R2
Permeation %		
Regression	0.00001	0.767
Lack of fit	0.208	
Content uniformity		
Regression	0.00005	0.774
Lack of fit	0.65064	

*P* is probability and R2 is regression coefficient.

### Regression model equations for CQAs

**Fig. 3:**
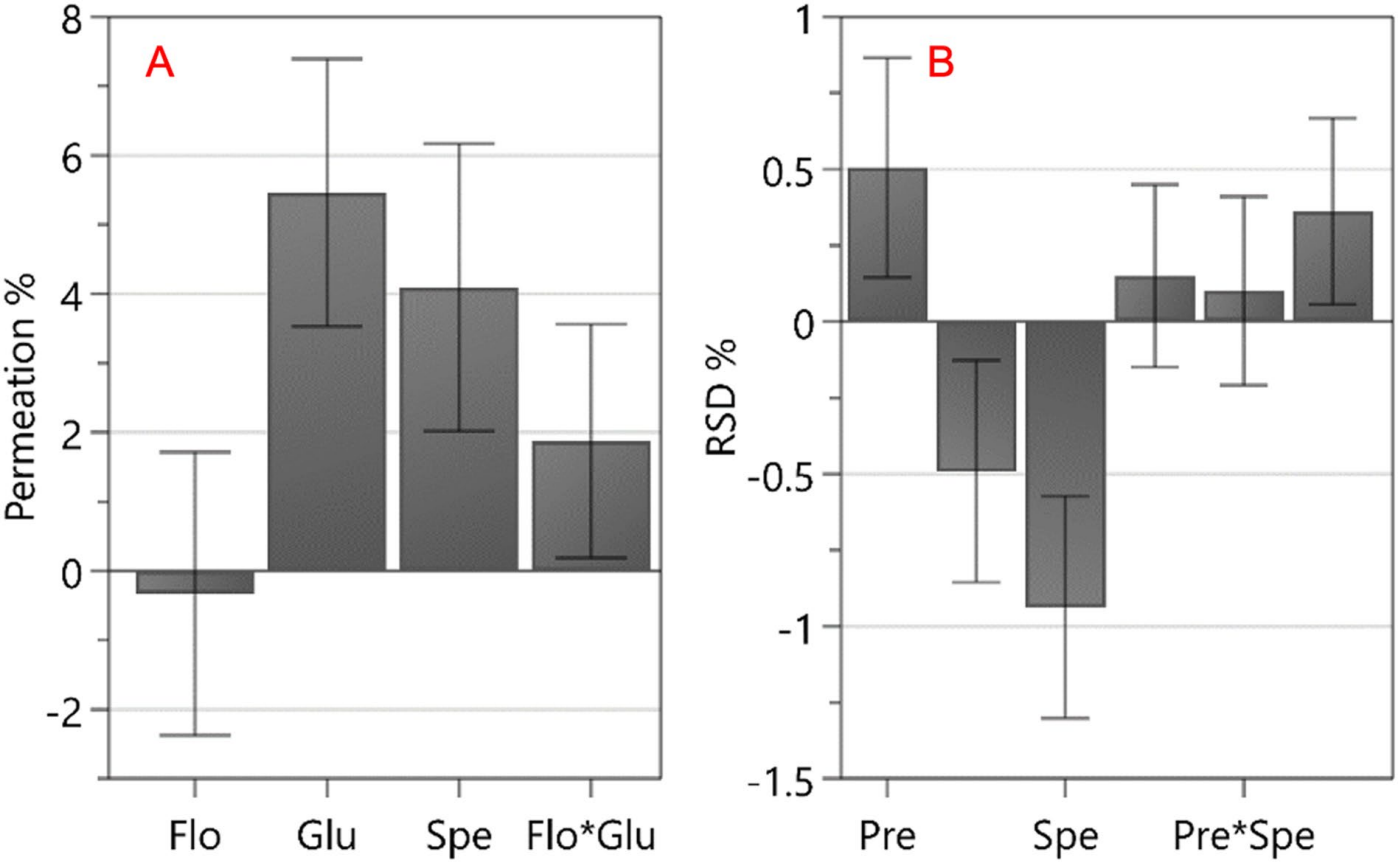
Regression coefficient plot of significant isothermal dry particle coating process model terms for the CQAs. (**A**) % permeation in 60 min, (**B**) % RSD of content uniformity.

After establishing the significance of the CQAs within the model, an investigation of the regression coefficients for all model terms was conducted to identify significant terms per CQA. This was investigated through regression coefficient plots, as depicted in Fig. 3. In these plots, the length of the bar represents each coefficient and signifies the magnitude of its effect on the response variable, while the direction of the coefficient indicates whether it exerts a positive or negative impact. A coefficient is deemed significant when the confidence intervals do not intersect at zero^35^.

The coefficient plot lists the significant coefficients, referred to as linear terms that are responsible for the main effects of CPPs. Subsequently, the following coefficients, denoted as interaction terms, reveal significant interactions among these factors. In this context, the size of the coefficient reflects the actual effects, while the confidence interval represents the presence of noise within the model, shown in Fig. 3.

In Fig. 3A, the analysis revealed that the primary factors influencing permeation percentage at 60 min were the concentration of L-glutamic acid and the speed (rpm) of the dry particle coater drum. Additionally, there was an interaction between flow rate and L-glutamic acid concentration, which significantly impacted permeation at 60 min. Figure 3B highlights that three CPPs along, one interaction among them which had an impact on the content uniformity in formulations. Pre-process time, process time, and the speed (rpm) of the dry particle coater drum were significant factors, along with an interaction between process time and speed (rpm).

Model equations for the investigated responses, generated from the regression coefficients, are presented in Eqs. 1 and 2. Insignificant terms (example quadratic terms) that did not compromise the hierarchy of the model were removed from the model equations.

1 $${Y}_{1}=44.8754+14.0811\,{X}_{4}+10.9087\,{X}_{5}+6.24105\,{X}_{3}{X}_{4}$$

2 $${Y}_{2}=3.02752+1.29385\,{X}_{1}-1.24592\,{X}_{2}-2.29596\,{X}_{5}+1.12108\,{X}_{2}{X}_{5}$$

where Y_1_ represents the percentage permeation of vancomycin released at 60 min, Y_2_ denotes content uniformity measured as RSD. The CPPs X_1_ through X_5_ correspond to pre-processing time (minutes), process time (minutes), flow rate (litres/minute), L-glutamic acid concentration (%), and drum speed (RPM), respectively.

The value of each coefficient indicates the magnitude of its impact on the responses (CQAs), a higher coefficient value indicates a more pronounced effect exerted by the CPP. The sign of the coefficient provides information on whether the impact is positive or negative. A positive sign associated with L-glutamic acid (X_4_), for instance, indicated that increasing the L-glutamic acid concentration increased the amount of vancomycin that permeated at 60 min. Whereas, for content uniformity, a negative sign associated with speed (rpm) (X_5_) indicates that a reduced speed decreased the RSD value, thus improving content uniformity.

### Effect of critical process parameters on permeation of vancomycin at 60 min

The iDPC comprises of a high-speed rotating drum, where increasing the drum’s rotational speed proportionally increases the centrifugal force acting on the powder formulation. Within the drum, a hollow rod emits a nitrogen gas stream that generates a drag force, fluidising the powder that has accumulated along the drum wall due to the centrifugal force.

To confirm that the observed enhancement in vancomycin permeation was not due to disruption of the TR146 cell layers, transepithelial electrical resistance (TEER) values were measured before and after permeation study. No statistically significant differences were observed, and TEER values remained within the expected range, consistent with previously reported values^33^. This indicates that the integrity of the TR146 cell layers was maintained throughout the permeation studies.

**Fig. 4:**
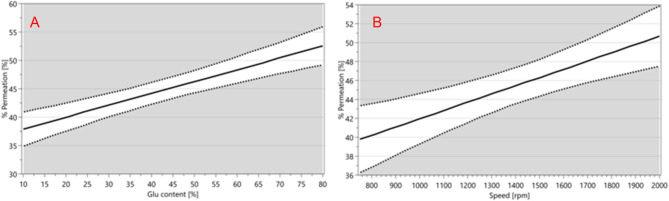
(**A**) Main effect plot of L-glutamic acid concentration illustrating the positive impact of increase in concentration on the quantity of vancomycin that permeated at 60 min. (**B**) Main effect plot showing that increasing the speed of the iDPC drum increased the permeation of vancomycin at 60 min.

As mentioned, two factors individually exhibited a positive impact on the permeation of vancomycin. Among the CPPs, the increase in L-glutamic acid concentration exerted the most significant influence, shown in Eq. 1. Following was the increase of the drum speed within the isothermal dry particle coater, as depicted in Fig. 4.

**Fig. 5:**
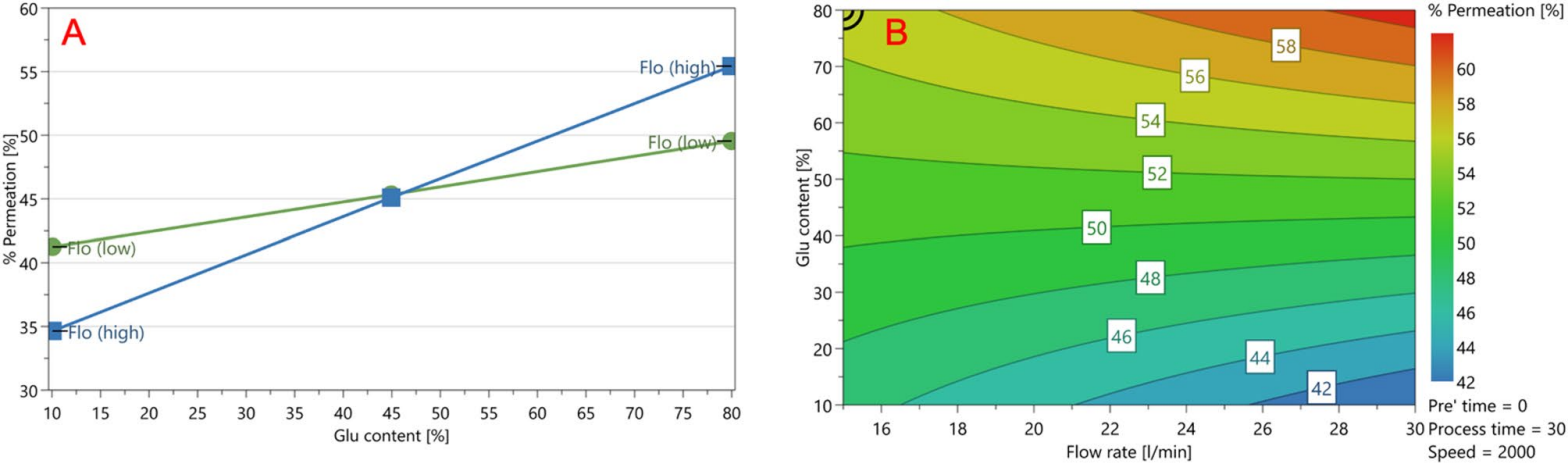
(**A**) The interaction plot showing the interaction point between flow rate (l/min) and L-glutamic acid concentration. (**B**) Contour plot illustrating the impact of increased flow rate and L-glutamic acid concentration on the quantity of vancomycin that permeated at 60 min.

In addition, the regression equation (Eq. 2) showed a positive interaction between flow rate and L-glutamic acid concentration that increased the quantity of vancomycin that permeated at 60 min, the interaction plot can be seen in Fig. 5A.

The observed positive impact on permeation of vancomycin by increasing the L-glutamic acid concentration is likely attributed to the formation of ion-pair neutral complexes^24^. According to the mass action principle, an excess of L-glutamic acid counterions drives the equilibrium towards the formation of these complexes by saturating the charged sites on vancomycin and reducing the likelihood of ion-pair dissociation^36^. Additionally, the increase in permeation may be due to a more complete surface coverage of vancomycin particles with L-glutamic acid particles, further decreasing ion-pair dissociation^37^. As a result, a more neutral, lipophilic complex is generated that can more readily permeate cell membranes. Supporting this mechanism, Iyire et al. (2016) demonstrated that introducing a counterion to insulin significantly enhanced the transcellular permeation of insulin across buccal cell layers^20^. Therefore, the significant increase in permeation observed with higher glutamic acid concentrations is likely attributed to both the enhanced stabilisation of ion-pairs and the associated reduction in the net charge of the complex, leading to improved permeation.

Increasing the speed of the rotational drum within the iDPC exhibited a positive relationship with permeation of vancomycin at 60 min, as indicated in Eq. 2. Increasing the drum speed leads to a rise in relative centrifugal force (RCF), which in turn may promote a more efficient coating process of the vancomycin particles with L-glutamic acid particles. When the drum speed in the iDPC is increased, the powders inside the drum experience stronger mechanical forces, primarily in the form of increased RCF. As RCF increases, particles are propelled towards the drum wall at a greater velocity, which in turn promotes deagglomeration by overcoming the cohesive forces (e.g. van der Waals or electrostatic forces) that typically hold powder aggregates together^38,39^. The elevated drum speeds also increase the frequency and intensity of particle-to-particle and particle-to-drum wall interactions, promoting the breakup of agglomerates into smaller, more uniformly distributed particles. This may allow for a more complete coating of L-glutamic acid particles on vancomycin particles, therefore leading to a more uniform and stable ion-pair formation^40^.

Figure 5A and B illustrates the positive interaction between flow rate and L-glutamic acid concentration on the quantity of vancomycin permeated at 60 min. This enhancement could be attributed to the combined effect of increased flow rate and excess L-glutamic acid, potentially inducing a degree of fluidisation. Fluidisation promotes the suspension of particles in a dynamic, fluid-like state, thus enhancing the probability of particle-particle interactions between the smaller L-glutamic acid particles and the larger vancomycin particles^41,42^. The presence of a greater number of finely dispersed L-glutamic acid particles significantly increases the available surface area, creating more contact points for vancomycin adsorption^43^. In a fluidised system, particle mobility is significantly enhanced and agglomeration is minimised, further amplifying the available surface area and facilitating robust surface interactions^44^. When L-glutamic acid particles are present in excess, these surface interactions become more pronounced, facilitating the robust adsorption of L-glutamic acid particles onto vancomycin particles. This closer association may lead to a more homogeneous blend, potentially promoting ion-pair formation and, in turn, increasing the quantity of vancomycin that permeated at 60 min^28,45^.

To quantitatively interpret the enhancement in L-glutamic acid coating onto vancomycin with increasing nitrogen flow rate, the theoretical number of ‘guest’ particles required to form a monolayer on each ‘host’ particle can be calculated. Using the established geometric relationship (Eq. 3) between the volumetric mean diameter (VMD) of vancomycin (51.64 μm) and L-glutamic acid (2.35 μm), it can be approximated that around 2,100 l-glutamic acid particles are theoretically required to create a single, closely packed monolayer on each vancomycin particle^46,47^. Where ${N}_{theoretical}$ is the theoretical loading capacity of ‘guest’ particles that can occupy the ‘host’ surface, ${d}_{host}$ is the diameter of the ‘host’ particle, and ${d}_{guest}$ is the diameter of the ‘guest’ particle.

3 $${N}_{theoretical}=\frac{4({d}_{host}+{d}_{guest})^{2}}{{d}^{2}}$$

However, whether those ~ 2100 l-glutamic acid particles actually collide and adhere depends strongly on the fluidising force within the iDPC. As the flow rate increases (from 15 to 22.5 l/min and up to 30 l/min), the resulting gas velocity rises significantly, increasing the drag force (${F}_{drag}$) exerted on each L-glutamic acid particle. This force can be approximated using the drag force equation (Eq. 4)^48^.

4 $${F}_{drag}\approx \frac{1}{2}\,{C}_{d}{\rho}_{gas}A{v}^{2}$$

where ${C}_{d}$ is the drag coefficient, ${\rho}_{gas}$is the density of nitrogen, $A$ is the projected area of a guest particle, and $v$ is the local gas velocity^49^. Doubling the flow rate approximately doubles the gas velocity, therefore quadrupling the drag force^50^. Under these higher flow conditions, a significantly higher proportion of L-glutamic acid particles remain suspended rather than agglomerating or adhering to the iDPC drum wall. This increased particle suspension promotes higher collision frequency with vancomycin particles, thereby enhancing the proportion of L-glutamic acid that successfully coats vancomycin particles.

This mechanism clarifies the positive interaction observed (Fig. 5A) between nitrogen flow rate and L-glutamic acid concentration on vancomycin permeation. At lower gas velocities (flow rates ≤ 15 l/min), excess L-glutamic acid particles tend to agglomerate or adhere to the sides of the drum wall, limiting interaction with vancomycin. In contrast, higher gas velocities (flow rates ≥ 22.5 l/min) can establish an effective nitrogen “curtain”, dispersing L-glutamic acid particles evenly through the drum. This consistent dispersion enhances particle interactions, significantly improves the extent of coating, promotes ion-pair formation, and yields a more neutral, lipophilic complex that will more readily permeate the buccal mucosa^28^.

### Effect of critical process parameters on content uniformity

**Fig. 6:**
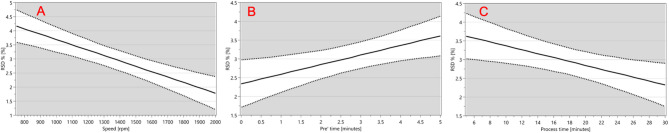
(**A**) Main effect plot illustrating that increasing drum speed reduced the RSD % (**B**) Main effect plot illustrating that increasing pre-processing time increase the RSD % (**C**) Main effect plot illustrating that increasing process time reduced RSD %.

Content uniformity was evaluated by RSD, with an acceptable target of ≤ 5%^30^. The regression Eq. (2) revealed that three factors (drum speed, pre-process time, and process time) and one interaction term (process time and drum speed) significantly impacted the content uniformity, as shown in Fig. 6. Among these, the most significant factor contributing to reduced RSD (i.e., improved content uniformity) was an increase in speed of the iDPC drum. The next most significant factor was pre-process time, which exhibited a positive effect on RSD (worsening content uniformity), while process time showed a negative effect (improving content uniformity).

The decrease in RSD correlated with an increase in drum speed, where the lowest RSD (0.93%) was recorded at the maximum speed tested (2,000 rpm), and the highest RSD (6.29%) at the lowest speed tested (750 rpm). This suggests that higher speeds enhanced content uniformity by dispersing fine particles more effectively and improving the overall homogeneity. A key factor behind this is the rise in RCF at higher drum speeds: as the RCF increases, finer particles are more thoroughly dispersed, reducing aggregation and helping achieve a more even distribution. This improved homogeneity is critical for batch to batch consistency and meeting pharmacopeial standards^12^.

**Fig. 7:**
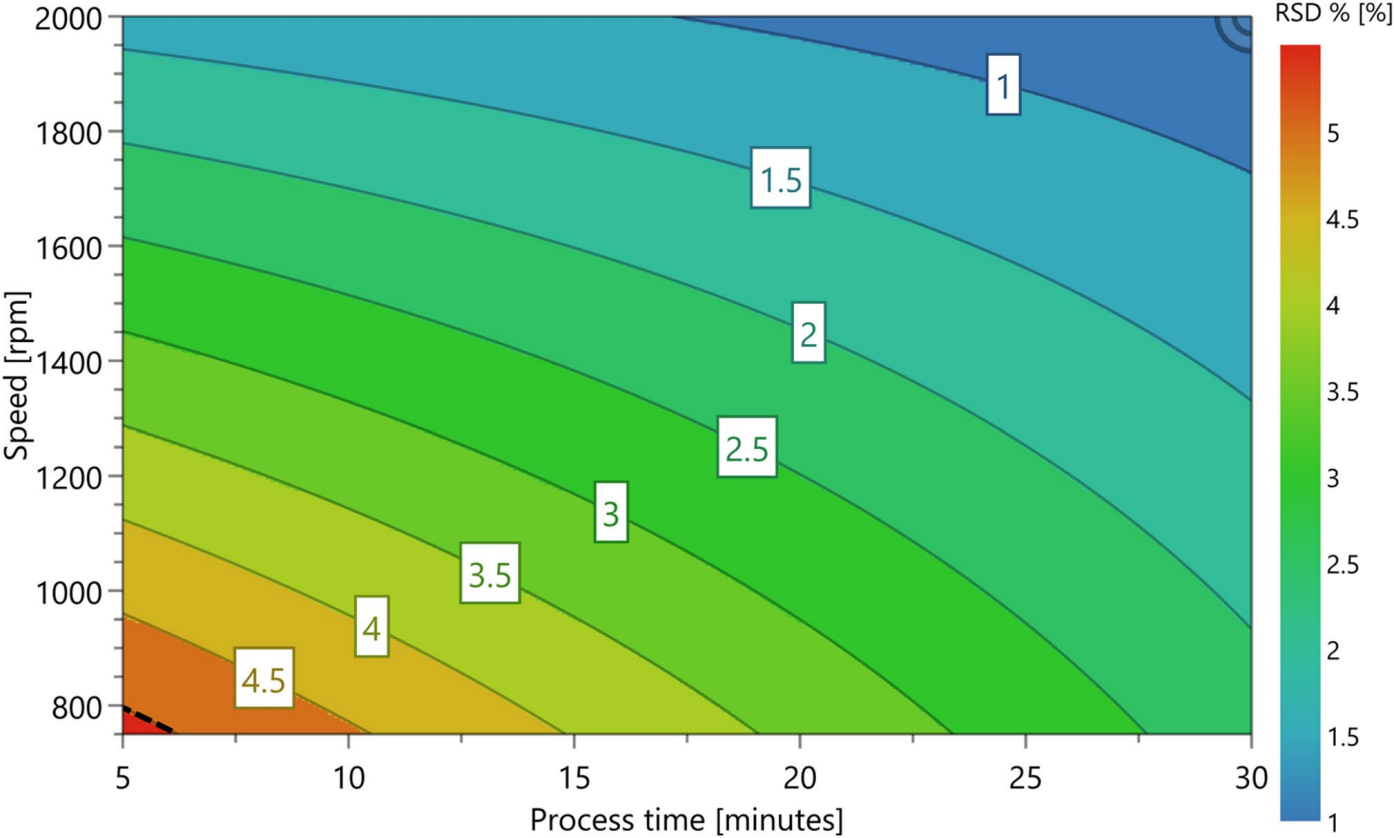
Contour plot illustrating the interaction of process time and speed and the associated impact on RSD %. As process time and speed (RPM) increases the RSD % decreases.

Figure 7 illustrates the interactive relationship between drum speed and process time. Prolonged processing at high speeds likely fosters continuous deagglomeration and extensive particle interaction, inhibiting the formation of segregated domains within the blend. This sustained process ensures that both vancomycin and L-glutamic acid are well-distributed across the entire formulation, further improving the content uniformity^51^.

Additionally, higher speeds facilitate more efficient particle adhesion, particularly in dry particle coating processes, where the uniform distribution of ‘guest’ components onto ‘host’ particles is essential^52^. The increased kinetic energy imparted by higher speeds may enhance particle-particle interactions, leading to better adhesion and a more consistent formulation^53^. In contrast, lower speeds may not provide sufficient energy for effective deagglomeration, resulting in non-uniform particle distribution and higher RSD values^54^. These findings suggest that optimising process parameters, particularly drum speed and processing time, is critical for achieving optimal content uniformity. Maintaining an appropriate balance between centrifugal force, processing time, and particle interaction dynamics can ensure a homogenised formulation.

A key mechanistic understanding of how the iDPC drum speed and processing time influence content uniformity can be derived by examining the rotational kinetic energy (${E}_{rot}$) imparted to powder bed within the iDPC, as shown in Eq. 5^55^.

5 $${E}_{rot}=\frac{1}{2}\,I,{{\upomega}}^{2}$$

where $I$ is the moment of inertia of the powder bed and ${\upomega}$ is the angular velocity, which directly relates to the iDPC’s drum speed. At higher drum speeds, the angular velocity increases substantially, leading to a substantial rise in kinetic energy available for particle-particle and particle-wall collisions within the drum.

As a result, this enhanced kinetic energy facilitates effective disruption of cohesive agglomerates by overcoming interparticle cohesive forces^55^. As processing time increases, the powder blend is exposed to sustained collisions and shear forces, progressively improving particle dispersion and homogeneity. The combination of prolonged processing time with increased drum speed creates a cumulative effect, whereby particles experience continuous, high-energy interactions. These sustained, intensified mechanical forces not only accelerate the breakdown of particle agglomerates but also help maintain consistent particle distribution throughout the powder bed. Consequently, this positive interaction significantly improves content uniformity by ensuring thorough and uniform blending.

### Design space creation

**Fig. 8:**
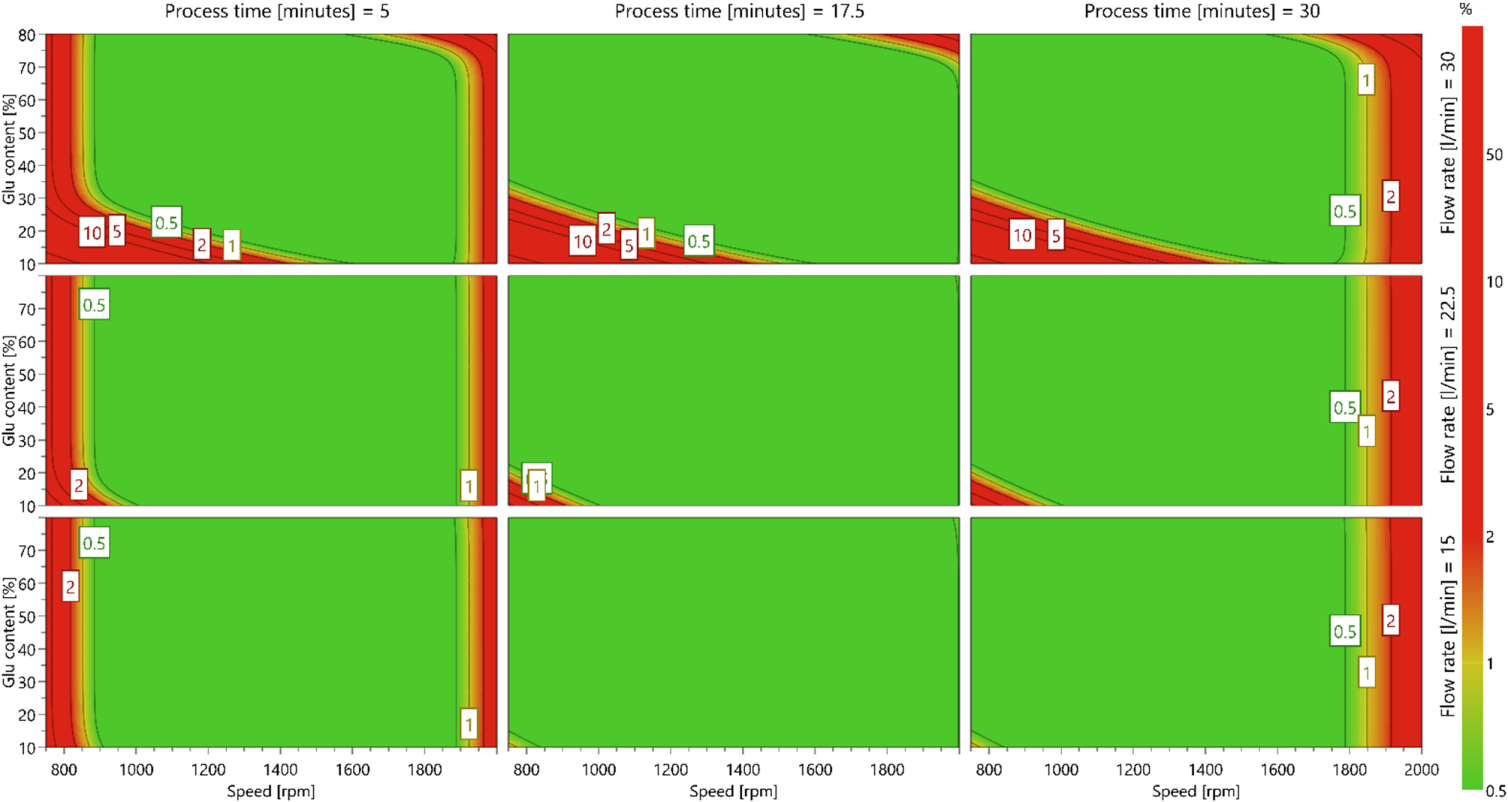
4D design space plot for vancomycin permeation percentage and RSD %, axis 1 is speed (750–2000 rpm), axis 2 is L-glutamic acid content (10%, 45% and 80%), axis 3 is process time 5–30 min), axis 4 is the flow rate (15 l/min, 22.5 l/min, and 30 l/min), and the constant is pre-process time and set to 2.5 min. The acceptance level is set to 1%. Simulations are set to 10,000/point. The green regions indicate a 0% chance of formulation failure, and areas that are red have a percentage of failure higher than 1%—the contour level labels indicate the probability percentage of failure.

The analysis conducted throughout this DoE enabled the plotting of a 4D design space for the iDPC, depicted in Fig. 8. This design space provides a visual representation of the probability of formulation failure to reach the required specifications. Regions depicted in green indicate a 0% chance of failure, while those in red denote varying degrees of failure percentages with the maximum being 10% in this design space^56^. The design space specifications had been set with RSD (%) less than 5%, aligning with the acceptable standards of the BP, and permeation of vancomycin through TR146 buccal cell line greater than 40% at 60 min^30^.

Figure 8 illustrates that iDPC as a manufacturing process provides a wide design space with low probability of formulation failure with the pre-set specifications. This underscores the capability of this manufacturing process to consistently produce formulations meeting desired specifications while allowing for potential deviations^10^. This design space offers several benefits in manufacturing pharmaceutical products, including the ability to streamline regulatory processes; as any minor adjustments within the design space would not require additional regulatory filings, thus enabling continuous process improvement creating a more efficient and cost-effective process^10,11^.

However, there was some observed predictive formulation failure (up to 10%) for formulations processed with low drum speed, high flow rate, and low L-glutamic acid concentrations. This increase in formulation failure is predominantly attributed to the formulations not achieving above 40% permeation of vancomycin. This observation aligns with the regression coefficient plot, shown in Fig. 3, which indicates that formulations containing lower L-glutamic acid concentrations exhibit reduced vancomycin permeation.

The primary factor contributing to this reduced permeation is likely due to the insufficient concentration of L-glutamic acid to facilitate stable ion-pair formation with vancomycin. An ion-pair complex can enhance drug permeability by neutralising charge and increasing lipophilicity, however, when the concentration of the counter-ion (L-glutamic acid in this case) is too low, there are not enough counter-ions to form a sufficient number of stable ion-pairs^57,58^. Consequently, a larger proportion of vancomycin remains in its charged, hydrophilic state, which limits its ability to permeate across the buccal membrane^59,60^.

## Conclusion

In this study, a QbD approach was used to evaluate the impact of CPPs of an iDPC on the CQAs of vancomycin formulations, specifically to enhance the permeation of vancomycin through TR146 buccal cell layers and to achieve an acceptable content uniformity aligning with Pharmacopeial standards. Through a comprehensive risk assessment, the selected CPPs included: pre-process time, process time, flow rate, speed of the rotational drum, and the concentration of L-glutamic acid. The results indicated that increasing the L-glutamic acid concentration, nitrogen flow rate, and drum speed positively impacted the permeation percentage of vancomycin which may be attributed to the drag force generated by the nitrogen curtain, facilitating the fluidisation and contact between fine and coarse particles. On the other hand, an increase in drum speed and process time achieved formulations within the acceptable content uniformity range. The resulting design space for the iDPC illustrates a wide design space indicating a low probability of formulation failure and underscoring the robustness of this manufacturing approach to consistently meet the desired quality criteria. These findings demonstrate that isothermal dry coating can be used to process macromolecules and create effective ion-paired formulations which can modulate drug permeability across buccal tissue and content uniformity.

## Materials and methods

The TR146 cell line was obtained from Public Health England. Vancomycin was purchased from Stratech, USA. Trifluoroacetic acid (HPLC grade) and L-glutamic acid, were purchased from Sigma-Aldrich, UK. Hanks’ balanced salt solution (HBSS), foetal bovine serum (FBS), Ham’s F-12 nutrient mix and trypsin-EDTA were obtained from Gibco^®^ Lab., UK. Gentamicin and penicillin/ streptomycin were obtained from Bio Sera, UK. Acetonitrile HPLC grade, absolute ethanol, Corning CoStar 12 mm diameter insert, and 12 well 0.4 μm polycarbonate membrane tissue culture treated polystyrene plates, were purchased from Fisher Scientific, UK. All water used was Ultrapure (Type 1) Direct-Q 3 UV and autoclaved.

### Screening studies

Previous work conducted identified L-glutamic acid as a suitable amino acid to enhance the permeation of vancomycin, potentially forming an ion-pair with vancomycin without disrupting the TR146 cellular integrity^8,20^. The QTPP was defined to increase the permeation of vancomycin through the TR146 buccal cell layers and achieve appropriate content uniformity, with a RSD of less than 5%. To establish the CQAs and CPPs, a comprehensive risk assessment was completed.

### Particle size analysis

Laser diffraction was employed to measure particle size using a Sympatec HELOS/BR particle size analyser equipped with a RODOS dry dispersing system with VIBRI/I feeder (Clausthat-Zellerfield, Germany). Approximately 0.5 g of sample was placed on to the VIBRI/L feeder tray and dispersed through a RODOS disperser with 3 bars of pressure. The volume mean diameter (VMD) of each sample was detected on the HELOS/BR set at a measuring range of 0–175 μm. All powders were measured in triplicate (*n* = 3), and the software calculate the 10% (D10), median (D50), 90% (D90) particle sizes and the volume mean diameter (VMD).

### Preparation of formulations using an isothermal dry particle coating process

A bench top, commercially available iDPC was used to prepare 1 g powder blend formulations that consisted of vancomycin and L-glutamic acid at varying concentrations. Vancomycin and L-glutamic acid were added to the dry particle coating drum. Each formulation was manufactured under a pre-selected set of conditions such as speed, processing time, and nitrogen gas flow (Table 4). Subsequently the formulation was retrieved from the dry particle coating drum using a brush.

### High performance liquid chromatography (HPLC) assay

HPLC method development of vancomycin was adapted from Hu et al.^22^. The Agilent 1220 Infinity II LC system with UV/fluorescence detector was used, utilising an Eclipse Plus column (C18 3.5 μm 4.6 × 150 mm). An isocratic elution was employed using a mobile phase consisting of acetonitrile and 0.1% trifluoroacetic acid (TFA), 13:87 [v/v]. The UV detection was set to 281 nm, the flow rate was 0.6mL/min, the injection volume was set to 5µL. Using this protocol, vancomycin eluted at approximately 8 min. This method was validated using ICH guidelines.

#### TR146 cell culture procedures

TR146 cells were grown and maintained in 75 cm T-flasks in Ham’s F-12 cell culture media with the addition of 50mL foetal bovine serum (FBS), 2.5mL of 1% penicillin-streptomycin, 10mL of 2mM glutamine, 1mL of gentamicin (10 mg/mL), and 1mL of amphotericin B (250 µg/mL). The cells were incubated at 37 °C, 5% CO_2_ and 95% air. The medium was changed every 2–3 days and when 90% confluency was reached, cells were passaged using 5mL of 50% Trypsin-EDTA in HBSS solution and seeded onto 12 well transwell inserts at a density of 24,000 cells/cm^2^. Passage number 12–15 were used for these experiments.

#### In vitro transbuccal permeation studies

 Permeability studies were conducted as described by Nielsen and Rassing^61^ at 37 °C and 140 rpm in an orbital plate shaker. Formulation solutions containing 450–500 µg/0.5mL HBSS and in the presence of amino acids with concentration ranging from 50 to 100 µg/0.5mL were used, with the pH of the solutions measured before and after the permeation study. Cells on the transwell were rinsed twice with HBSS (37 °C) by adding 0.5mL to the apical chamber and 1.5mL to the basolateral chamber, and TEER measured. After 2 h (of experiments), cells were rinsed with 0.5mL HBSS and equilibrated for 30 min after which TEER values were measured. All samples were analysed for vancomycin content by HPLC.

### Transepithelial electric resistance (TEER)

The ohmic resistance of cells grown on transwell inserts was measured using an EVOM3 (Epithelial Volt/Ohm Meter) with chopstick electrodes. The electrodes were placed erect, such that the longer electrode touched the basolateral chamber, while the shorter electrode touched the apical membrane chamber. TEER, which reveals the integrity of the cellular layers, was calculated from triple readings from replicate transwells (*n* = 9).

### Content uniformity

Three samples of each formulation containing an equivalent quantity of vancomycin were accurately weighed and dissolved in 10mL of Ultrapure (Type 1) Direct-Q 3 UV water, in a volumetric flask. The samples were then filtered using 0.4 μm syringe filter and quantified by HPLC. The degree of homogeneity of the mixture was measured by the RSD of the mean content value.

### Design of experiments (DoE)

The DoE was statistically designed using MODDE software version 13.0.2.34314 (Umetrics Inc., NJ, USA). A CCF design (star distance: 1) was selected to evaluate the interactive effects of the variables associated with the iDPC (pre-process time, process time, flow rate, L-glutamic acid content, and speed). Pre-processing time (minutes) refers to the time for which the ‘host’ particles were processed alone in the iDPC drum. This step was performed to evaluate whether the iDPC induces deagglomeration of ‘host’ particles, potentially enhancing content uniformity in the final formulation. Process time (minutes) denotes the total duration which both ‘host’ and ‘guest’ particles were processed together in the iDPC drum. Flow rate (L/min) represents the rate of nitrogen flow. Speed (rpm) refers to the rotational speed of the iDPC drum. Experiments were carried out according to the run order prescribed by MODDE and are depicted in Table 4.

**Table 4:** The CCF design worksheet with CPPs and the total number of runs completed.

Experiment number	Pre-process time (min)	Process time (min)	Flow rate (l/min)	L-glutamic acid content (%)	Speed (RPM)
N1	0	5	15	10	2000
N2	5	5	15	10	750
N3	0	30	15	10	750
N4	5	30	15	10	2000
N5	0	5	30	10	750
N6	5	5	30	10	2000
N7	0	30	30	10	2000
N8	5	30	30	10	750
N9	0	5	15	80	750
N10	5	5	15	80	2000
N11	0	30	15	80	2000
N12	5	30	15	80	750
N13	0	5	30	80	2000
N14	5	5	30	80	750
N15	0	30	30	80	750
N16	5	30	30	80	2000
N17	0	17.5	22.5	45	1375
N18	5	17.5	22.5	45	1375
N19	2.5	5	22.5	45	1375
N20	2.5	30	22.5	45	1375
N21	2.5	17.5	15	45	1375
N22	2.5	17.5	30	45	1375
N23	2.5	17.5	22.5	10	1375
N24	2.5	17.5	22.5	80	1375
N25	2.5	17.5	22.5	45	750
N26	2.5	17.5	22.5	45	2000
N27	2.5	17.5	22.5	45	1375
N28	2.5	17.5	22.5	45	1375
N29	2.5	17.5	22.5	45	1375

### DoE model analysis

Generated results were used to fit the PLS model, after which model verification was carried out to ensure its validity and reproducibility through sequential elimination of the insignificant runs using distance to model plots, while evaluating the effect of the elimination on the model using lack of fit and summary of fit plots.

ANOVA analyses for total variations of the responses were carried out (variances attributed to regression model and variances obtained from residuals and replicate errors). The p-value for the significance of the regression coefficient was set to less than 0.05, while the model error or lack of fit was set to a value of greater than 0.5, indicating the insignificance of the model error.

A statistical model comprising the interaction terms was used to evaluate the responses, and it was displayed according to Eq. 6 for each significant response^12,62^:

6 $$y=\beta_{0}+\beta_{1}{x}_{1}+\beta_{2}{x}_{2}+\beta_{3}{x}_{3}+\beta_{12}{x}_{1}{x}_{2}+\beta_{23}{x}_{2}{x}_{3}\cdots+\epsilon$$

where ($y$) is the modelled response, ${\beta}_{0}$ is the arithmetic mean response of the runs; ${\beta}_{1}{,\beta}_{2},{\beta}_{3}$are the estimated coefficients for the main effects (${x}_{1},{x}_{2},x_{3},x_{4},x_{5}$), and the interaction terms (${x}_{1}{x}_{2}$, ${x}_{2}{x}_{3,}\dots$). $\epsilon$ is a constant. The final regression model was established through the evaluation of individual terms and the elimination of all insignificant terms. Once the model achieved significance, evaluation and prediction tools were employed to assess the impact of individual or interaction CPPs on CQAs. Subsequently the results were used to predict design space^62^.

## Supplementary Information

Below is the link to the electronic supplementary material.

Supplementary Material 1

## Data Availability

All data generated or analysed during this study are included in this published article and supplementary Information (Table S1).
