# Supplementary material for: Quality by design optimisation of isothermal dry particle coating for enhanced buccal permeation of vancomycin
Source: Sci Rep. 2025 Nov 25;15:45299. doi: 10.1038/s41598-025-29164-2 (PMC12749408; doi:10.1038/s41598-025-29164-2)
Supplement: Supplementary file 1 — Supplementary Material 1 [file 41598_2025_29164_MOESM1_ESM.docx]

Quality by Design Optimisation of Isothermal Dry Particle Coating for Enhanced Buccal Permeation of Vancomycin

Anthony Rajabi^1^, Affiong Iyire^1^, David Wyatt^2^, Jasdip Koner^2^, Afzal R Mohammed^1*^

^1^Aston Pharmacy School, College of Health and Life Sciences, Aston University, Birmingham, UK

^2^Aston Particle Technologies Ltd, Birmingham, UK

*Corresponding author

Aston Pharmacy School

Aston University

Birmingham

B4 7ET

Email: [a.u.r.mohammed@aston.ac.uk](mailto:a.u.r.mohammed@aston.ac.uk), [a.iyire@aston.ac.uk](mailto:a.iyire@aston.ac.uk)

Table S1 Experimental data for all formulations (N1–N29) from the Central Composite Face (CCF) design. Values show vancomycin permeation (%) at 60 minutes across TR146 buccal epithelium with standard deviation (SD), and content uniformity expressed as relative standard deviation (%RSD) (N=3).

| Formulation | % permeation at 60 minutes | SD | Content uniformity (%RSD) |
| --- | --- | --- | --- |
| N1 | 43.9 | 2.2 | 1% |
| N2 | 38.6 | 4.5 | 5% |
| N3 | 39.3 | 2.7 | 2% |
| N4 | 46.1 | 4.2 | 3% |
| N5 | 27.6 | 1.7 | 5% |
| N6 | 37.9 | 3.3 | 4% |
| N7 | 46.5 | 4.8 | 1% |
| N8 | 51.6 | 2.7 | 4% |
| N9 | 48.8 | 2.8 | 6% |
| N10 | 40.9 | 1.6 | 1% |
| N11 | 59.8 | 3.8 | 1% |
| N12 | 34.5 | 3.8 | 3% |
| N13 | 59.8 | 2.6 | 4% |
| N14 | 32.0 | 7.5 | 6% |
| N15 | 32.1 | 3.5 | 2% |
| N16 | 60.4 | 2.8 | 2% |
| N17 | 45.2 | 4.3 | 1% |
| N18 | 52.8 | 5.7 | 4% |
| N19 | 49.0 | 2.9 | 1% |
| N20 | 42.1 | 5.1 | 3% |
| N21 | 46.9 | 3.5 | 4% |
| N22 | 47.1 | 3.0 | 3% |
| N23 | 36.3 | 1.2 | 4% |
| N24 | 56.8 | 2.1 | 2% |
| N25 | 41.2 | 2.3 | 3% |
| N26 | 34.0 | 5.7 | 1% |
| N27 | 37.9 | 5.3 | 2% |
| N28 | 40.0 | 2.0 | 2% |
| N29 | 42.4 | 2.4 | 4% |
